# It Is in the Eye of the Beholder: Ocular Ultrasound Enhanced Monitoring of Neurotoxicity after CAR-T Cell Therapy

**DOI:** 10.3390/hematolrep15010001

**Published:** 2022-12-29

**Authors:** Juan Esteban Garcia-Robledo, Cristina Valencia-Sanchez, Molly G. Knox, Brent P. Goodman, Allison C. Rosenthal, Bhavesh Patel, Januario E. Castro

**Affiliations:** 1 Division of Hematology/Oncology, Mayo Clinic, Phoenix, AZ 85054, USA; 2 Department of Neurology, Mayo Clinic, Phoenix, AZ 85054, USA; 3 Department of Critical Care Medicine, Mayo Clinic, Phoenix, AZ 85054, USA

**Keywords:** immune effector cell-associated neurotoxicity syndrome, CAR-T cell therapy, ocular ultrasound, cancer therapy

## Abstract

Usually used in emergency settings, bedside sonographic measurement of optic nerve sheath diameter can aid in diagnosing elevated intracranial pressure. We report a case of a 26-year-old male hospitalized for CAR T-cell therapy with Axicabtagene Ciloleucel for treatment of relapsed diffuse large B-cell lymphoma, who developed progressive symptoms of immune effector cell-associated neurotoxicity syndrome. Fundoscopic examination suggested the presence of blurred optic disc margins. Bedside ocular ultrasound revealed wide optic nerve sheath diameters and bulging optic discs bilaterally. The patient had a ventriculostomy placed for monitoring and received treatment with steroids and mannitol, as well as tocilizumab. After 7 days in the ICU, the patient recovered with no evidence of long-term neurological deficits.

## 1. Introduction

Immunotherapy with chimeric antigen receptor (CAR) modified T-cells is a promising treatment for patients with refractory leukemia and lymphoma [1,2,3,4]. Toxicities include cytokine release syndrome (CRS) and immune effector cell-associated neurotoxicity syndrome (ICANS) [5]. These unique toxicities can be fatal, especially in cases of raised intracranial pressure (ICP) and cerebral edema [6,7]. Penissi et al. analyzed 102 patients that received CAR T-cell therapy, 53 with B-cell acute lymphoblastic leukemia (B-ALL), and 49 with diffuse large B-cell lymphoma (DLBCL). They found that ICANS was present in 50% of patients (55% in the B-ALL group and 45% in the DLBCL group) [8]. Other analyses have identified ICANS in up to 67% of patients with B-ALL and 62% of patients with DLBCL [9].

Various systems have been used for grading neurotoxicity. The most recent consensus criteria by the American Society for Transplantation and Cellular Therapy (ASTCT) include the 10-point Immune Effector Cell-Associated encephalopathy (ICE) score (a modified version of the CARTOX-10) [8], which evaluates orientation, naming, following commands, writing, calculation, level of consciousness, seizures, motor deficits and signs of increased ICP, using a 10 point-based system. Grades 1 (7–9 ICE) and 2 (3–6 ICE) are defined by the degree of encephalopathy. Grade 3 is defined by a more severe encephalopathy, seizures, or local edema on imaging (0–2). Grade 4 is defined as stupor or coma, ICE score of 0, prolonged or repetitive seizures, motor deficits, or signs of elevated ICP. Grade 5 describes death due to ICANS [5].

Due to the high frequency of neurotoxicity, close monitoring with a neurologic exam and noninvasive tools is imperative for prompt therapeutic interventions to avoid dire outcomes. Invasive or noninvasive methods can detect elevated intracranial pressures. The sonographic measurement of the optic nerve sheath diameter (ONSD) has been described as a sensitive and specific noninvasive method to detect increased ICP [10]. The optic nerve sheath contains the extension of the subarachnoid space.

The subarachnoid space surrounds the optic nerve and is circumscribed by the optic nerve sheath that is an anatomical extension of the dura mater around the nerve. In cases of increased ICP, the optic nerve sheath gets dilated due to the elevated cerebrospinal fluid (CSF) pressure [11]. ONSD measurement is performed using a linear probe placed on the closed upper eyelid, with the patient in the supine position. ONSD is measured 3 mm behind the globe in the two-dimensional mode [12]. Sonographic ONSD higher than 5.0 mm has been proposed as a cutoff for identifying increased ICP in adults [13].

We report a case of ICANS, monitored with ONSD ultrasonography, with a favorable outcome after aggressive treatment of intracranial hypertension.

## 2. Case Report

**Figure 1:**
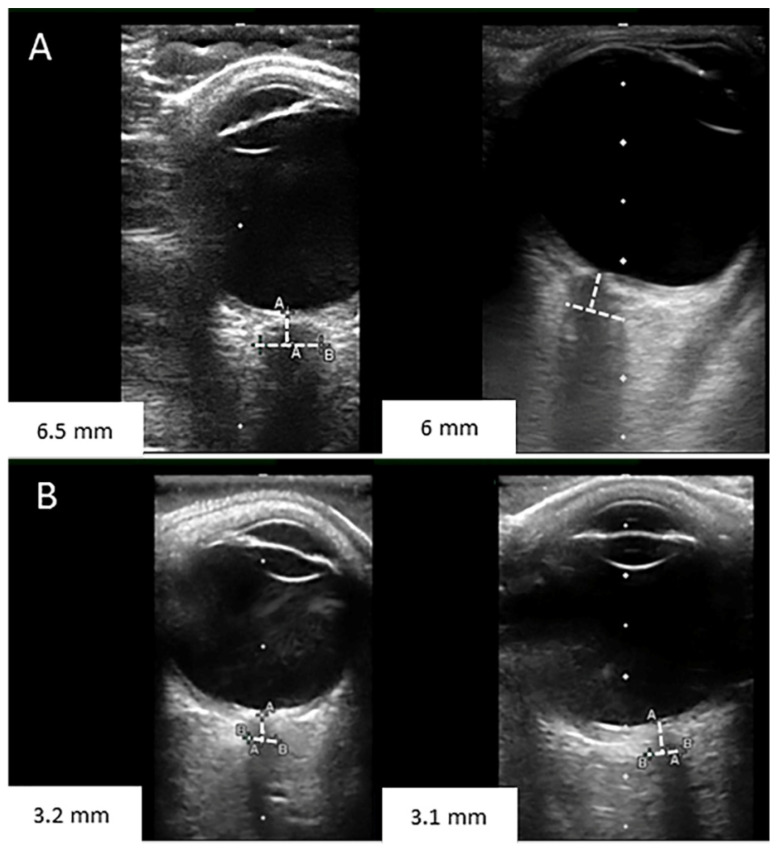
(**A**) Optic nerve sheath diameter measurements pre lumbar puncture were 6.5 and 6 mm. (**B**) Optic nerve sheath diameter measurements post lumbar puncture were 3.2 and 3.1 mm.

A 26-year-old male with refractory diffuse large B-cell lymphoma involving the mediastinum was treated with axi-cel CAR-T cell therapy (commercial autologous anti-CD19 CAR-T cell therapy). His neurological exam and brain MRI were normal before therapy. He was placed on seizure prophylaxis with levetiracetam per institutional guidelines. On the first day post-infusion, he developed fever and tachycardia consistent with grade 1 CRS. On day 4, he developed rapidly progressive ICANS that started with handwriting impairment and 7 h later became encephalopathic with an ICE score of 3. Examination revealed expressive aphasia, nystagmus, and mild tremor. Due to agitation, the funduscopic examination could not be reliably performed to assess for papilledema. Head CT was unremarkable. He received intravenous tocilizumab and dexamethasone 10 mg. Continuous EEG showed a moderate generalized slowing of background activity. Over the next 6 h, he became lethargic. Bedside ocular ultrasound was performed and ONSD was measured in the right optic nerve (6.5 mm) and the left optic nerve (6 mm) (Figure 1).

**Figure 2:**
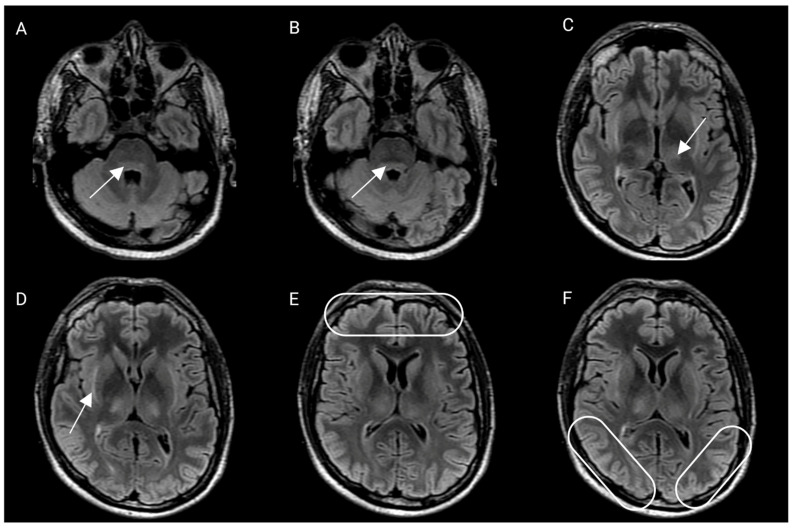
Brain magnetic resonance imaging, axial fluid-attenuated recovery sequence (FLAIR). Arrows and ovals show the areas described in each section. (**A**,**B**) There is a hyperintensity that shows a compromise of the dorsal pons at the level of the fourth ventricle, involving the nucleus of the VI and VII cranial nerves bilaterally. (**C**) There is a compromise of the thalamus at its ventromedial aspect, bilaterally. (**D**) Compromise of the subinsular external capsules. (**E**,**F**) There is evident cortical edema, most probably of cytotoxic origin.

He was intubated for airway protection. Lumbar puncture was performed with an opening pressure of 44 cmH_2_O. After removing 80 mL of cerebrospinal fluid, the closing pressure was 15 cmH_2_O. ONSD was measured again, obtaining a diameter of 3.2 and 3.1 mm for the right and left optic nerves, respectively. He was treated with high-dose methylprednisolone, hyperosmolar therapy, and sedation for grade 4 ICANS. An external ventricular drain (EVD) was placed for monitoring (first measure after placement was 3 cmH_2_O). A brain MRI showed increased T2-hyperintensity in the thalami and dorsal pons (Figure 2).

**Figure 3:**
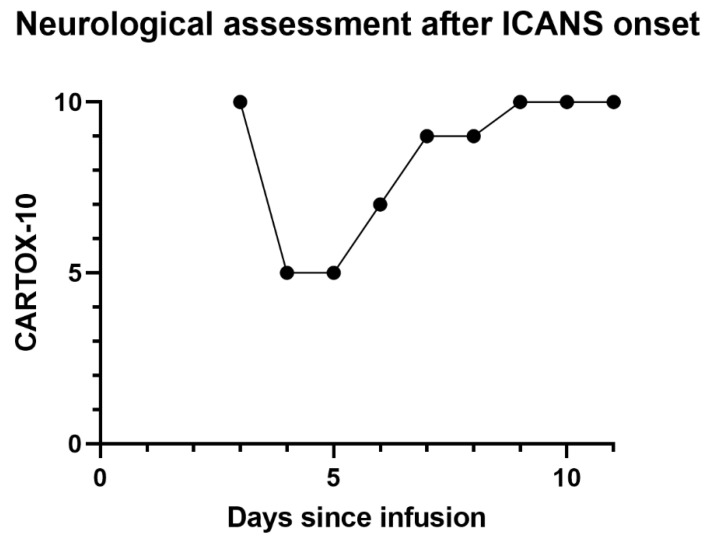
CARTOX-10 score since development of ICANS until discharge.

**Figure 4:**
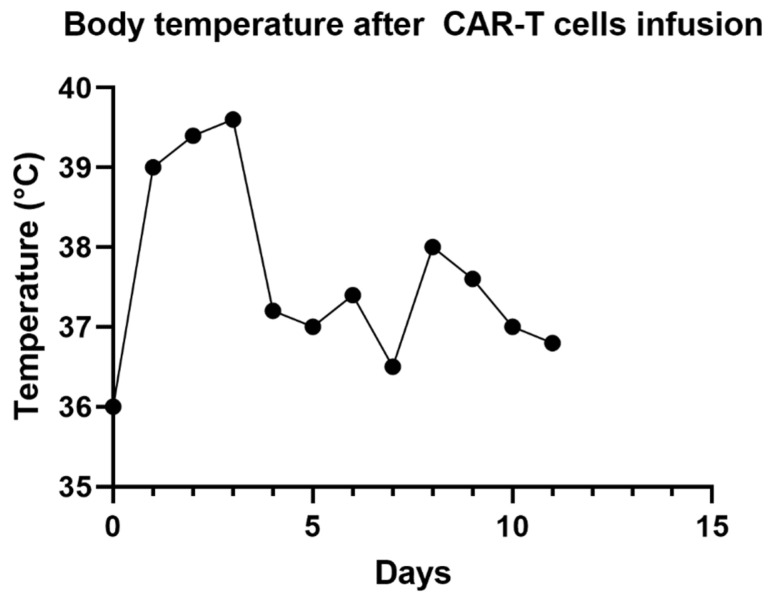
A timeline depicting changes in temperature during impatient management.

**Figure 5:**
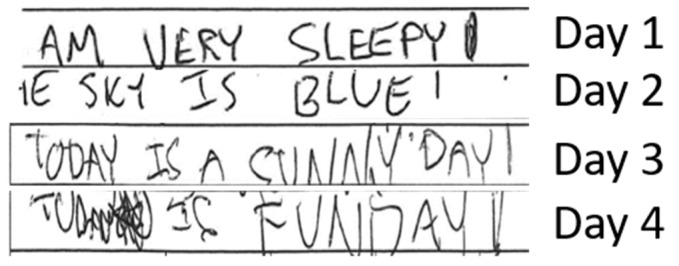
A timeline depicting changes in handwriting since CAR-T cell infusion.

**Figure 6:**
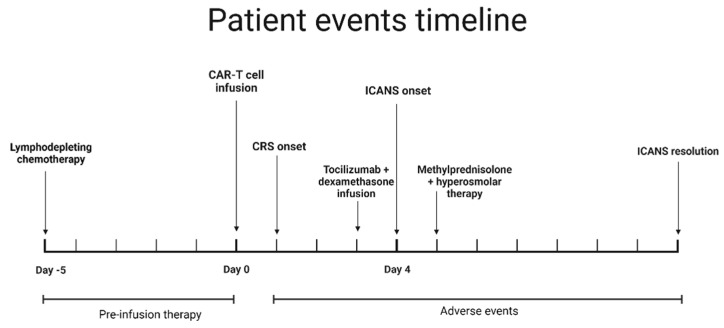
A timeline depicting the most important events in this patient’s case.

He received 3 doses of tocilizumab and 3 doses of methylprednisolone (1000 mg per dose). Serial ONSD measurements continued to remain low and were consistent with low ICP as measured by EVD. He was extubated 48 h after onset of neurotoxicity, and EVD was removed 2 days later. Neurotoxicity symptoms resolved one week after onset. Repeated MRI 3 weeks later showed resolution of the abnormalities. Lymphoma restaging one month after treatment revealed a complete response to therapy. A timeline depicting changes in temperature, ICP and CARTOX-10 can be seen in Figure 3, Figure 4 and Figure 5. Additionally, Figure 6 depicts a summary of the events in this case.

## 3. Discussion

Neurological toxicities have been observed in up to 77% of patients treated with CAR-T-cells [14], with grade 3–5 in 25–57% of patients [6]. The underlying pathophysiology of ICANS remains unclear, with possible mechanisms including an inflammatory process triggered by CAR-T-cells, with subsequent secretion of monocyte-derived cytokines like IL-1, IL-6, and GM-CSF, as well as a widespread endothelial activation with increased permeability of the blood–brain barrier (BBB). Studies of CSF from patients who developed severe forms of ICANS showed a 17-fold increase in CD14+ myeloid cells’ levels compared to patients with low-grade ICANS. Therefore, ICANS might be the result of systemic inflammation triggered by CAR-T cell infusion and activation, which leads to endothelial activation, BBB disruption, and inflammatory infiltration of central nervous tissues [15]. The most common neurological symptoms include encephalopathy, headache, tremor, aphasia, and focal weakness [14]. Expressive aphasia is the most specific first sign of severe neurotoxicity [5]. We reported a case of raised ICP with cerebral edema in which noninvasive monitoring of ICP with ONSD serial measurements allowed for rapid identification and initiation of aggressive treatment and monitoring of response with good correlation with invasive EVD-ICP measurements.

A meta-analysis by Cai et al. assessed the incidence and characteristics of fatal toxicity associated with CAR-T cell therapy. After analyzing 19 clinical trials with a total of 890 cases and 33 treatment-related deaths, they found that the most common causes of CAR T-cell therapy-related mortality were CRS (30.3%), nervous system disorders (18.2%) and infections (12%). Other causes included blood, cardiac, respiratory, gastrointestinal and hepatobiliary disorders [16]. Regarding nervous system involvement, cases of raised ICP with and without cerebral edema after CAR T-cell therapy have been reported^3^ and represent the most devastating sequelae of ICANS and the leading cause of neurologic CAR-T cell therapy-associated mortality [17].

Prompt recognition of intracranial hypertension after CAR-T cell therapy is necessary to mitigate adverse outcomes. However, assessment of papilledema in patients with encephalopathy using fundoscopy is challenging and, most of the time, inaccurate [18]. Additionally, obtaining imaging studies such as CT or MRI may be delayed due to logistical reasons and the hemodynamical/neurological stability of the patient, which can create critical delays to initiate lifesaving therapeutic interventions. Moreover, the gold standard assessment for ICP using invasive lumbar puncture opening pressure may be contraindicated due to coagulopathy, thrombocytopenia, or clinical instability, and poses a risk of complications such as herniation and bleeding [19].

## 4. Conclusions

Our case demonstrates sonographic measurement of ONSD as a bedside, noninvasive and reliable method to monitor intracranial pressure and ICANS in patients undergoing CAR-T-cell therapy. This tool can be used for effective monitoring of critically ill patients that require early and aggressive management. Bedside ocular ultrasound can be used serially for early detection of increased ICP after cellular therapy enabling early and aggressive management of cerebral edema which can be a devastating neurologic complication of CAR-T cell therapy. We encourage the development of large cohort studies that address this technique to better understand its impact in CAR-T cell therapy monitoring and similar events.

## Data Availability

Not applicable.
